# Efficacy of *tuina* in patients with chronic neck pain: study protocol for a randomized controlled trial

**DOI:** 10.1186/s13063-018-3096-3

**Published:** 2019-01-17

**Authors:** Zhiwei Wu, Lingjun Kong, Qingguang Zhu, Pengfei Song, Min Fang, Wuquan Sun, Hao Zhang, Yanbin Cheng, Shanda Xu, Guangxin Guo, Xin Zhou, Zhizhen Lv

**Affiliations:** 1 Tuina Department, Yueyang Hospital of Integrated Traditional Chinese and Western Medicine, Shanghai University of Traditional Chinese Medicine, Shanghai, 200437 China; 2 Institute of Tuina, Shanghai Institute of Traditional Chinese Medicine, Shanghai, 200437 China; 3 Shanghai University of Traditional Chinese Medicine, Shanghai, 201203 China

**Keywords:** Chronic neck pain, Efficacy, Randomized controlled trial, *Tuina*, traditional Chinese massage

## Abstract

**Background:**

Chronic neck pain (CNP) is a common and disabling musculoskeletal disorder in developing and developed countries. Previous studies have shown that *tuina* and traditional Chinese massage are effective treatments for patients with CNP. However, there is little evidence to support the use of one intervention over the other. The aim of this study is to compare the effects of *tuina* and traditional Chinese massage in the treatment of pain and disability in patients with CNP.

**Methods/design:**

This is a multicenter, assessor- and analyst-blinded, randomized controlled trial with two parallel arms: a *tuina* group and a traditional Chinese massage group. A total of 356 eligible CNP patients will be randomly assigned to the groups in a 1:1 ratio. The intervention in the *tuina* group includes both structural and relaxation massage, while the traditional Chinese massage group will receive relaxation massage only. The interventions for both groups will last for 15 min and will be carried out three times a week for a period of 4 weeks. The primary outcome will be changes in the Northwick Park Neck Pain Questionnaire. Secondary outcomes will be measured by a visual analogue scale (VAS), the Neck Disability Index (NDI), and the 36-item Short-Form Health Survey (SF-36). The data will be analyzed at the baseline, at the end of the intervention, and during the 3 months of follow-up by repeated measures analysis of variance. The significance level is 5%. The safety of *tuina* and traditional Chinese massage will be evaluated after each treatment session. The results of this trial will help clarify the value of *tuina* and traditional Chinese massage as treatments for CNP and will highlight any differences in the efficacy of the treatments.

**Discussion:**

The purpose of this trial is to determine whether *tuina* is more effective than traditional Chinese massage in adults with CNP. This trial will, therefore, contribute to providing a solid foundation for clinical treatment of CNP, as well as future research in massage therapy.

**Trial registration:**

Chinese Clinical Trial Registry, ChiCTR-INR-17013763. Registered 8 December 2017.

**Electronic supplementary material:**

The online version of this article (10.1186/s13063-018-3096-3) contains supplementary material, which is available to authorized users.

## Background

Chronic neck pain (CNP) is a serious public health and socioeconomic problem worldwide. Studies have shown that the overall prevalence of CNP varies from 0.4% to 86.8% (mean 23.1%), while the 1-year incidence of CNP in a high-risk population (office and computer workers) ranged from 10.4% to 21.3%, which is greater than for manual workers [1]. CNP is characterized by activity limitations, pain, dizziness, anxiety, and insomnia [2]. A cross-sectional study showed that health-related quality of life was also negatively correlated with prevalent neck pain [3], and the existence of neck pain was also a predictor of poor future physical health-related quality of life in a longitudinal study [4]. Moreover, CNP causes a significant socioeconomic impact, not only due to direct medical costs, but also due to indirect health-care costs and loss of productive capacity [5, 6].

The pathophysiology of CNP is not well established due to difficulties in localizing the source of the pain. Potential causes of CNP include, but are not limited to, changes in the spinal disc structure with aging and degeneration, as well as changes in local concentrations of cytokines such as matrix metalloproteinase, phospholipase A2, nitric oxide, IL-1β, IL-6, C-reactive protein, and tumor necrosis factor α (TNF-α) [7]. Specific conditions contributing to CNP include degeneration, inflammation, infective and neoplastic causes, metabolic bone diseases, referred pain, psychogenic pain, trauma, congenital disorders, exposure to tobacco from direct smoking, physical activity, and repetitive and sedentary work positions [8].

Depending on the tailored treatment strategies, various treatment modalities are available for CNP including medication (non-steroidal anti-inflammatory drugs and serotonin reuptake inhibitors) and interdisciplinary rehabilitation, physical therapy, spinal injections, and surgical interventions [9–11]. Although pain-relieving medication is most frequently used to alleviate CNP, long-term use of non-steroidal anti-inflammatory drugs is limited due to the risk of side effects and patient intolerance [9]. Complementary therapies such as massage, acupuncture, neck exercises, mechanical traction, herbal medicine, cupping, electrotherapy, and exercise are well accepted as alternative treatments for CNP due to their positive effects and safety profile [12–14]. There have been many trials on the use of traditional Chinese massage to treat CNP. However, there is considerable controversy regarding its efficacy due to poor treatment precision, variations in treatment, inadequate sample sizes, low methodological quality, and subclinical dosing regimens of trials to date.

*Tuina* is commonly defined as an ancient healing art using fingers and strength [15]. In treating CNP with *tuina*, the spinal joints are adjusted with the lightest strength for as short as possible to obtain the best clinical curative effect. In recent years, its efficacy has been confirmed by many trials [12, 14]. Thus, we designed a randomized controlled trial (RCT) to investigate whether *tuina* is more effective than traditional Chinese massage in treating CNP. This trial will, therefore, contribute to providing solid clinical evidence on the efficiency of *tuina* and traditional Chinese massage for CNP, as well as future research in massage therapy.

## Methods/design

### Study design

**Fig. 1:**
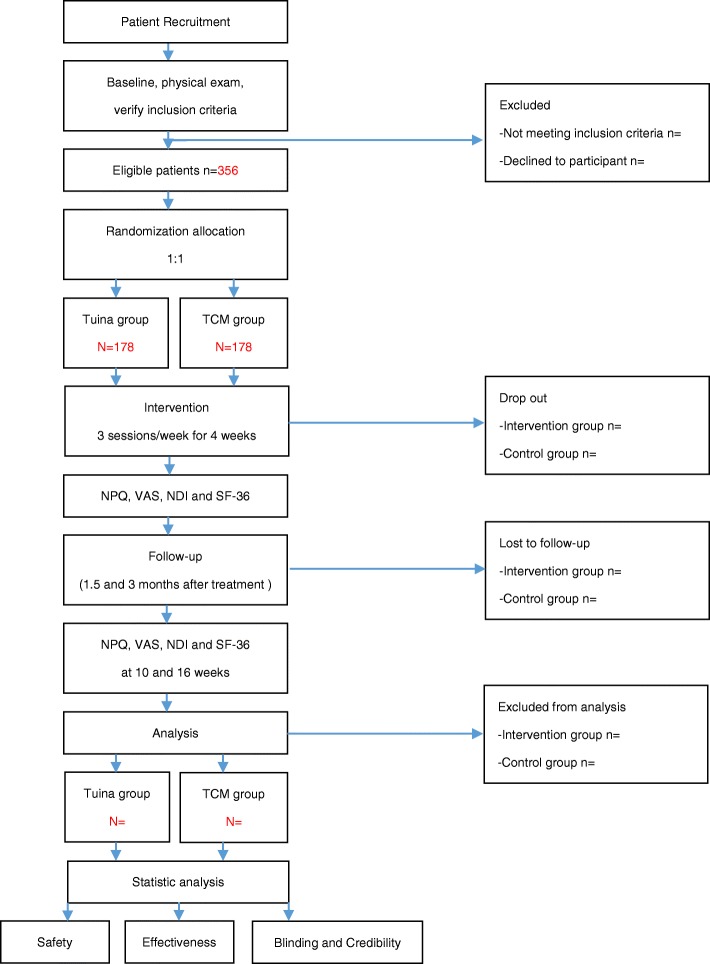
Flow chart of the study. A total of 356 participants will be randomized to the two groups. The interventions will last for 15 min and will be carried out three times a week for 4 weeks. The study period will be consisted of the baseline, 2 weeks of treatment, after treatment, 1.5 and 3-month follow-up. NDI Neck Disability Index, NPQ Northwick Park Neck Pain Questionnaire, SF-36 36-item Short-Form Health Survey

**Fig. 2:**
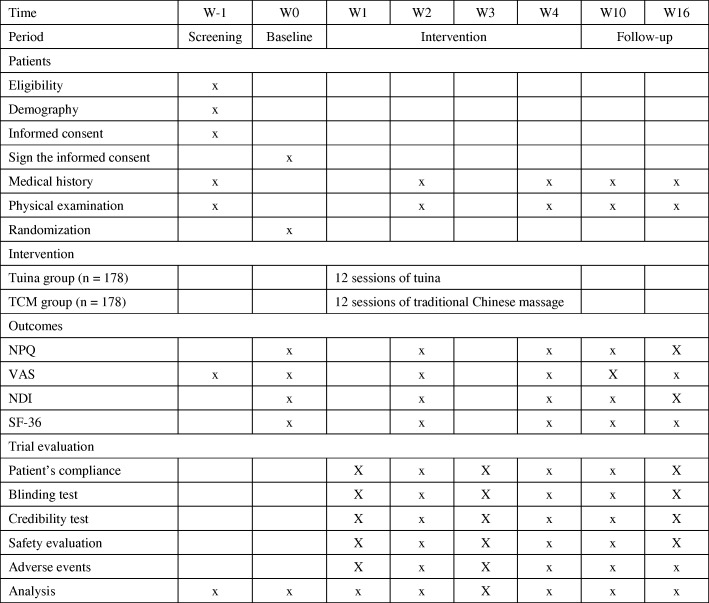
Study schedule showing time points for enrollment and assessment. NDI Neck Disability Index, NPQ Northwick Park Neck Pain Questionnaire, SF-36 36-item Short-Form Health Survey, VAS visual analogue scale, W-1 screening before enrollment, W0 baseline assessment, W2 assessment after the sixth treatment, which is in the second week, W4 assessment after the 12th treatment, which is in the fourth week, W10 assessment 10 weeks after the first treatment, W16 assessment 16 weeks after the first treatment

This is a multicenter, assessor- and analyst-blinded RCT with two parallel arms: a *tuina* group and a traditional Chinese massage group. The study protocol has been approved by the Regional Ethics Review Committee of Yueyang Hospital of Integrated Traditional Chinese and Western Medicine affiliated with Shanghai University of Traditional Chinese Medicine (project number: 2017–028). It has been registered in the Chinese Clinical Trial Registry (ChiCTR-INR-17013763). A total of 352 eligible CNP patients will be randomly assigned to the groups in a 1:1 ratio. Written informed consent will be provided by all patients. Outcome assessment and statistical analyses will be performed by independent researchers who are blinded to patient assignment. The study design is illustrated in the flow chart in Fig. 1 and the study schedule is presented in Fig. 2.

The main treatment center will be Yueyang Hospital of Integrated Traditional Chinese and Western Medicine affiliated with Shanghai University of Traditional Chinese Medicine, which will enroll 116 participants. Three other treatments centers—Sixth Hospital of Shanghai, Shuguang Hospital affiliated with Shanghai University of Traditional Chinese Medicine, and Jiading District Hospital of Traditional Chinese Medicine—will each enroll 80 participants.

### Participants and recruitment

Eligible participants are patients diagnosed with CNP according to the clinical guideline for traditional Chinese medicine (Criteria of Diagnosis and Therapeutic Effect of Diseases and Syndromes in Traditional Chinese Medicine, ZY/T001.1–94). Patients whose neck pain has persisted without relief for over 2 weeks such that it impairs their daily activities at work or elsewhere will be informed of this trial. If a patient expresses interest, a clinical trial communicator will describe the trial to them and ask whether they would like to join it. If the answer is yes, a face-to-face interview will be conducted in the reception room for clinical research subjects in the four hospitals. Patients who meet the inclusion criteria and do not meet the exclusion criteria can join once they sign the consent form.

We publicized the trial to potential participants in four ways: 1) by approaching patients with neck pain admitted to an outpatient department or inpatient ward in each center, 2) recruitment posters at each center and nearby community centers, 3) the official microblog and WeChat platforms of each center, and 4) printed adverts in newspapers.

### Inclusion criteria

Participants who meet all the following criteria can be enrolled:

1. With neck pain as their main symptom for at least 3 months but less than 2 years
2. Episodes of pain lasting for over 30 min, and frequency of pain occurring at least once per month
3. Pain intensity of more than 3 points as measured on a visual analog scale (VAS) at the time of recruitment
4. No massage or other relevant treatment has been received within the 7 days prior to study entry
5. Aged 20–60 years, male or female
6. Able and willing to comply with the intervention and follow-up evaluation
7. Agree not to receive other relevant treatments during the period of treatment (including muscle relaxants)
8. Able to provide written informed consent

### Exclusion criteria

Participants meeting any of the following criteria will be excluded:

1. History of cervical intervertebral disc herniation accompanied by nerve symptoms; neck trauma; surgical neck fracture; spinal disease; infectious inflammatory autoimmune diseases; congenital vertebral anomalies; or compression fractures
2. With a severe primary disease such as cardiovascular, cerebrovascular, liver, or kidney disease
3. Unable to communicate or provide self-care due to a psychiatric or psychological disorder related to severe neurosis or due to dementia
4. Women who are pregnant or lactating
5. Partial injury in acute phase or with local skin damage
6. Duration of neck pain or neck stiffness episodes of less than 0.5 h
7. Pain episodes have occurred for less than 3 months or more than 2 years
8. Failure to adhere to the treatment at the prescribed time
9. Use of massage or other relevant treatment within 7 days prior to study entry
10. Participating in other clinical trials
11. Use of any other treatment (drug or non-drug)

### Dropout criteria

Participants who do not complete the clinical protocol for the following reasons should be considered as dropped out:

1. The patient quits (poor efficacy or adverse reactions)
2. Loss to follow-up
3. Researchers remove the patient (poor compliance, complications, or serious adverse events)

The trial communicator will communicate in-depth with patients who want to withdraw from the trial because of poor efficacy or have no time to promote retention.

### Comprehensive suspension criteria

The trial will be suspended if:

1. The investigators discover a significant safety problem
2. The therapeutic effect is poor (We will asses the therapatic effect at week2)
3. There is a major mistake in the plan
4. The sponsor has a huge problem in funding or management

### Randomization

In this trial, the Department of Science and Technology of Yueyang Hospital will generate the randomization sequence using a random number generator (SPSS 21.0, SPSS Inc., Chicago, IL, USA). Random numbers will be placed in opaque envelopes, numbered sequentially, and sent to a therapist. The envelopes are opened in numerical order to determine the allocation for participants who pass the screening test. The unmasking is performed by the therapist after the baseline questionnaire is filled in, after the physical examination, and after checking the exclusion criteria, so that the study administrator, the therapist, and the study participant are blinded to the group assignment until after all baseline data have been collected.

### Blinding

Patients will not be informed of the type of treatment that they will receive. Outcome assessors, data managers, and the statistician will be blinded in this trial and do not share study information with each other. To preserve masking, only the massage therapists will have access to treatment allocation. However, they must learn how to use the blinding method to communicate with participants to ensure treatment blinding. The blinding procedure will be operated until the data are locked.

### Interventions

Participants will receive 12 sessions of treatment over a period of 4 weeks. Treatment will be performed by a senior therapist who has studied acupuncture and massage and has held a practitioner’s license for more than 10 years. They must have received professional training in *tuina* and massage. They must pass a test to ensure consistency of study methods before participating in the trial. The participants will be asked to rest for 15 min before treatment. A constant room temperature of 23 to 25 °C will be maintained to ensure the patients are comfortable and relaxed during treatment. Subjects will be advised to lie in the prone position on the treatment couch. The interventions will be performed three times a week. Each session will last for 15 min. Neck function will be assessed at baseline as well as at 2, 4, 10, and 16 weeks after the baseline assessment.

### *Tuina* group

In this arm of the study, the therapist will administer a two-step protocol intended to ease neck pain and improve neck functional activity disorders by releasing the soft tissue of the neck and shoulders, by inducing a state of general relaxation while addressing specific structural issues determined by the clinician to be likely to contribute to the patient’s CNP, or by promoting *qi* movement (which according to traditional Chinese medicine theory activates blood circulation). The specific protocol used is described below.

#### Step one: local manipulation

Patients are instructed by the therapist to lie in the lateral position and to relax their mind and body naturally. Neck pain conditions can be carefully examined prior to treatment. Tender tissue, trigger points, contracted muscle tissue (knots), and nodules are identified for further treatment. The therapist first relaxes the soft tissue from the neck and shoulders to the upper back for 5 minutes. The pain point is the *a-shi* point in acupuncture theory. It is generally recognized as reflecting the underlying condition and is frequently manipulated to stop pain. The pain point is pressed in the neck region in a direction perpendicular to the erector spine for 5 min. This step will be performed to resolve adhesions and to increase general circulation with muscle pressing, stripping, and deep tissue kneading manipulation. The manipulation will be gradually intensified and enlarged. It is intended to unblock *qi* stagnation, remove blood stasis by separating adherent fascicles, and resolve contracted muscle nodules. The amount of force used is determined by the patient’s *deqi* sensation, which is often described as a dull pain, heaviness, numbness, or soreness, and commonly regarded as an indicator of manipulation efficacy in acupuncture and *tuina* [16–18].

#### Step two: neck structural rectification

Neck structural rectification is performed after the above procedures have relieved the tension in muscles and soft tissues. The patient will be instructed to lie in the lateral position with the affected side up. The therapist presses one thumb on the anterior intercondylar nodule of the lordotic segment of the lesion while the palm of the same hand holds the lower jaw. The other thumb is pressed on the upper or lower vertebral articular while the palm holds the occipital. The operator first stretches the patient’s neck longitudinally for a moment, then corrects the sagittal shift of the cervical spine by adjusting the diseased segment lightly with both thumbs. At the end of the treatment, the therapist relaxes the patient’s neck by gentle traction and twisting forces with both hands. All manipulations will last 5 min.

### Traditional Chinese massage group

The traditional Chinese massage group will receive relaxation therapy for neck pain. The protocol has two steps.

#### Step one: click on the acupuncture point manipulation

The therapist instructs the patient to sit, and then presses and kneads GB20, GB21, SI14, LI15, LI11, and LI4 for 1 min each.

#### Step two: local manipulation

The therapist applies a roll and *nafa* (characterized by a pinch and lift up) to relax the muscle and soft tissue of the neck and shoulders. This procedure will last for 9 min.

### Allowance of concurrent treatment of patients

All treatments for CNP are banned during the trial, including oral muscle relaxants, narcotics, analgesics, surgery, drug injections, acupuncture, and physical therapy. They may receive any treatment that is not related to neck pain. Any change in concurrent treatment will be recorded at every visit.

### Outcome measurements

Four well-recognized self-report tools will be used to measure the outcomes. The Northwick Park Neck Pain Questionnaire (NPQ) will be used as the primary outcome measure. A VAS, Neck Disability Index (NDI), and Short Form 36 (SF-36) will be used as secondary outcome measures. Neck pain-related dysfunction, pain, and quality of life will be assessed at six time points: screening, baseline (after enrollment), weeks 2 (intervention session), 4 (intervention session), 10 (follow-up session), and 16 (follow-up session). We will measure the study participant’s previous experience of the interventions, their expectations for recovery, and their expectation of the importance of the allocated therapy in their recovery (just after the unmasking and before the intervention starts).

### Primary outcome measurement

The NPQ has been widely used in research and has been shown to have high validity and reliability for neck pain measurement [19, 20]. It comprises nine items on the degree and duration of pain, symptoms including numbness, sleep, social activities, and quality of life. The highest score is 100 and high scores indicate severe damage due to neck pain. The efficacy is measured as the percentage improvement in the NPQ. If the efficacy is greater than 30%, the treatment is considered to be successful. The NPQ efficacy rate is the percentage of participants for whom treatment was successful. It will be assessed using repeated longitudinal analysis per group.

### Secondary outcome measurements

#### VAS

The intensity of pain in the cervical region is measured with a 10-cm VAS, for which 0 means “absence of pain,” while 10 represent “the worst pain imaginable.” The patients will be asked: “How much pain do you have at this moment?” To extract the data, the researcher measures the distance between the start of the scale (absence of pain) and the mark set by the participant. The VAS will be assessed using a repeated longitudinal analysis. The VAS has been shown to be a valid and reliable outcome measure [21–23] and the test–retest reliability has proven to be good (intraclass correlation 0.92) [24].

#### NDI

The change in neck function will be evaluated using the NDI, which is the most frequently used questionnaire to evaluate cervical pain and functional limitations in daily life. It consists of 10 questions about pain intensity, personal care, lifting, reading, headaches, concentration, work, sleeping, driving, and recreation. Each question is answered on a scale from 0 to 5. The total score ranges from 0 to 50 points, with higher scores indicating more severe symptoms. At least 8 of the 10 sections must be answered for the score to be calculated. The NDI is reliable and valid [25–28]. The NDI will be assessed using a repeated longitudinal analysis.

#### SF-36

The SF-36 questionnaire assess the correlation between health-related quality of life and various factors [29, 30]. It consists of 36 questions grouped in 8 domains: vitality (4 items), physical functioning (10 items), bodily pain (2 items), general health (5 items), physical role (4 items), emotional role (3 items), social functioning (3 items), and mental health (5 items). For each domain, scores range from 0 to 100 and higher scores reflect a better quality of life. The SF-36 will be assessed using a repeated longitudinal analysis.

### Safety evaluation

At each visit, researchers will evaluate adverse events (AEs), which are defined as unexpected or unfavorable responses that occur during or after treatment. In this trial, AEs are defined as events that (1) lead to hospitalization, (2) prolong a department (Outpatient department or inpatient wards) or hospital stay, (3) cause a disability, (4) hinder ability to work, or (5) are a threat to life. For any AE, regardless of whether it is related to the interventions, treatment should be terminated immediately. The patient should receive any remedial treatment and the AE will be reported to the relevant responsible units and ethical committees to determine whether the patient should drop out of the trial. All patients with an AE will be followed up until the event has been resolved or the patient’s condition has become chronic or stable.

### Follow-up

To evaluate the short-term efficacy, long-term efficacy, and the safety of the interventions, we will follow up participants for 3 months after the trial. During the 3 months of unsupervised follow-up, no participants will undergo special therapy with the exception of routine cervical care. At weeks 10 and 16, the outcome assessor will telephone participants to investigate the recurrence of their neck pain. Patients can also inform the assessors of their clinical symptoms and AEs face to face or by email, text message, or WeChat at the relevant time points.

### Blinding test and credibility test

Questionnaires on blinding will be completed at weeks 2, 4, 10, and 16. The success of these blinding strategies will be appraised at the end of the study. The credibility rating for both treatments will be evaluated using a credibility test at week 2 and at the end of the treatment with a seven-point scale developed by Vincent et al. The participants will rate the scores (0 = very low confidence and 6 = very high confidence) for the following four questions:

- How confident do you feel that this treatment can alleviate your chronic neck pain?
- How confident would you be in recommending this treatment to a friend who suffered from a similar complaint?
- How logical does this treatment seem to you?
- In your opinion, how successful could this treatment be in alleviating other complaints?

### Data collecting and monitoring

Demographic and baseline characteristic data will be collected by screeners when the patients are recruited. Clinical outcomes, questionnaire-based assessment of treatment effects and neck physiological function, and details of adverse events will be recorded by outcome assessors in case report forms (CRFs). Completed CRFs are reviewed by a four-person steering committee, which is composed of the principals of the four research centers, and submitted to the data administrators, who are independent from the research team and blinded to group allocation, for data entry and management using an Excel database. All data administrators have data analysis qualifications and are trained uniformly. To ensure the accuracy of the data, two data administrators independently enter the information and proofread it. If there are issues with the information in the CRF, the data administrators can fill out a query sheet and give it to the steering committee. The data administrators can then modify the data according to any revisions made by the steering committee. After confirming that the database is correct, the steering committee, data administrators, and statistician will lock the database.

This trial uses an electronic data management system. The system integrates electronic CRFs, data entry, data locking, data exporting, and system contingency plans, which enable scientific tracking and real-time monitoring of the test data.

The Department of Science and Technology in Yueyang Hospital, which is not taking part in the study, will be responsible for monitoring the data. The CRFs, protocol compliance, data management, treatment administration, and AEs will be monitored independently during the study.

### Statistical analyses

The analysis set will consist of a full analysis set (FAS), a per protocol set, and a safety set. The safety set will include any participants who were randomly assigned and received at least one massage treatment. The FAS will include data indicating that the treatment was as close to intention to treat as possible. In addition to meeting the criteria of the safety set, to be included in the FAS, the participants must have been evaluated for the primary outcome at least once. To be included in the per protocol set, participants must receive more than nine sessions of treatment (75%). The FAS will be used in the main analysis. For missing data, we will analyze the underlying reason and use an imputation adjustment approach, and the last observation carried forward analysis will be used to handle the missing data. After the main analysis, a sensitivity analysis will be performed by comparing the results from the per protocol analysis and the intention to treat analysis to evaluate the impact of missing data on the trial results.

Data normality will be tested through visual inspection of histograms. According to the results of the homogeneity and normality analysis, parametric statistics (Tukey test) or non-parametric statistics (Wilcoxon rank sum test) will be used for the within- and between-group analyses. Descriptive statistics will be used to compare demographic and baseline information and to evaluate the credibility of the groups. If an adjustment for possible baseline incomparability is needed, a covariance analysis will be performed. Efficacy is measured at five time points. Therefore, a repeated measures analysis of variance will be used to investigate the effect of treatment, time, and interaction terms between treatment groups versus time. Two-sided paired *t* tests will use within-group comparisons (comparing baseline to follow-up). A chi-squared test or a Fisher’s exact test will be performed to determine categorical variables between groups and adverse effects, which will be recorded and described as a frequency and percentage. Fisher’s exact test will be used if a cell has an expected value of less than 5 that is equal to or more than 20%. All statistical analyses will be performed with the Statistical Package for Social Sciences (SPSS, version 21.0, SPSS Inc., Chicago, IL, USA) by statisticians who are independent of the research team and blinded to the group allocation. The confidence interval will be established at 95%, and the significance level at 0.05.

### Sample size calculation

The expected efficacy rate of the *tuina* group measured by the NPQ is 95%. According to the literature, the efficacy rate for traditional Chinese massage is 85%. The sample size was calculated with a significance level of 0.05 and power of 0.80. According to the calculation in SPSS, the required sample size was 320. With a maximum dropout tolerance of 10%, 356 patients are needed for the trial, with 178 for each group across the four centers.

### Quality control

To maintain quality, quality control will be carried out throughout the trial. Monitoring information will be regularly submitted to the directors and will be retained for future reference. The steering committee, which is composed of the principals of the four research centers, is responsible for the coordination, development, and quality control of all the programs in the trial. All researchers are required to receive professional training on the trial method, study technique, and the method used for regular monitoring before participating in the trial. The researchers will be tested after training to ensure consistency of methods. Any modifications or corrections to the study protocol will be discussed by the steering committee and submitted to the ethics committee. Detailed records of changes will be kept.

## Discussion

Neck pain is one of the most challenging public health issues worldwide. Given its prevalence, the need to assess effective approaches for neck disorders is of prime importance. *Tuina* is one of the most important parts of traditional Chinese medicine and has contributed to the health of people in China for a thousand years. A considerable body of scientific evidence supports such therapies for the treatment of neck pain, but their efficacy has not been established. Therefore, it is important to provide strong evidence on the efficacy of *tuina* for CNP.

The present trial is a comparative effectiveness and safety study of *tuina* (intervention) and traditional Chinese massage (control) for pain relief and function recovery in patients with CNP. For a high-quality RCT, an appropriate control is crucial. In this trial, two questions must be addressed. First, is the *tuina* treatment effective for neck pain? Second, does *tuina* have a superior effect to traditional Chinese massage in treating neck pain? The *tuina* techniques used in this trial combine relaxation and structural massage methods, while traditional Chinese massage does not include joint adjustment. Since both pain intensity and physical function are subjective, we will use validated scales and questionnaires to assess the clinical outcomes. We evaluated three aspects of neck pain: pain, physical function, and quality of life. The NPQ will be used as the primary outcome. It measures neck pain, radiculopathy, and impairment of daily functions. The secondary outcomes are a VAS, which measures pain intensity in general settings, the NDI, which evaluates cervical pain and functional limitations in daily life, and the SF-36, which measures changes in health-related quality of life.

According to traditional Chinese medicine, good physical health depends on the circulation of *qi* and blood. The common causes of pain are stagnation of *qi* and stasis of blood. Moreover, other pathogenic factors such as phlegm and dampness can be identified as causative factors in a blockage. *Tuina* can relieve pain by promoting the local and systemic circulation of *qi* and blood by removing pathogenic factors. The *a-shi* point in traditional Chinese medicine is a reaction point for disease on the skin. Manipulation at an *a-shi* point can remove the local stasis of *qi* and blood and promote their circulation. Studies have shown that *tuina* may alleviate CNP by reducing inflammation and by repairing the damaged mitochondria of skeletal muscle [31].

The multi-center RCT design and methodological rigor of this trial will allow valuable and high-quality data to be collected to evaluate the efficacy of *tuina* and traditional Chinese massage for treating CNP. The trial will contribute to a solid foundation for the clinical treatment of CNP, as well as future research on *tuina* and massage therapy. The Standard Protocol Items: Recommendations for Interventional Trials (SPIRIT) Checklist shows the detailed information of the protocol items (Additional file 1).

### Study limitations

One of the major methodological difficulties inherent to studies evaluating physical interventions is blinding of participants. In this trial, *tuina* and traditional Chinese massage use different forms of manipulation, which is unfavorable to the blinding. Unmasking will be performed by the care provider after the physical examination, after checking the exclusion criteria, and after completion of the baseline questionnaires, so the study administrators, the therapists, and the study participants will be blind to the group assignment until after all baseline data have been collected.

### Trial status

This trial is recruiting patients now. Participant recruitment started in December 2017 and is expected to end in October 2018.

## Additional file

Additional file 1: SPIRIT 2013 checklist: recommended items to address in a clinical trial protocol and related documents. (DOC 126 kb)

## Abbreviations

AE: Adverse event
CNP: Chronic neck pain
CRF: Case report form
FAS: Full analysis set
NDI: Neck Disability Index
NPQ: Northwick Park Neck Pain Questionnaire
RCT: Randomized controlled trial
SF-36: 36-item Short-Form Health Survey
SPSS: Statistical Package for Social Sciences
VAS: Visual analogue scale
